# Mapping the Psychosocialcultural Aspects of Healthcare Professionals’ Information Security Practices: Systematic Mapping Study

**DOI:** 10.2196/17604

**Published:** 2021-06-09

**Authors:** Prosper Kandabongee Yeng, Adam Szekeres, Bian Yang, Einar Arthur Snekkenes

**Affiliations:** 1 Department of Information Security and Communication Technology Norwegian University of Science and Technology Gjøvik Norway

**Keywords:** information security, psychological, sociocultural, health care professionals

## Abstract

**Background:**

Data breaches in health care are on the rise, emphasizing the need for a holistic approach to mitigation efforts.

**Objective:**

The purpose of this study was to develop a comprehensive framework for modeling and analyzing health care professionals’ information security practices related to their individual characteristics, such as their psychological, social, and cultural traits.

**Methods:**

The study area was a hospital setting under an ongoing project called the Healthcare Security Practice Analysis, Modeling, and Incentivization (HSPAMI) project. A literature review was conducted for relevant theories and information security practices. The theories and security practices were used to develop an ontology and a comprehensive framework consisting of psychological, social, cultural, and demographic variables.

**Results:**

In the review, a number of psychological, social, and cultural theories were identified, including the health belief model, protection motivation theory, theory of planned behavior, and social control theory, in addition to some social demographic variables, to form a comprehensive set of health care professionals’ characteristics. Furthermore, an ontology was developed from these theories to systematically organize the concepts. The framework, called the psychosociocultural (PSC) framework, was then developed from the various combined psychological and sociocultural attributes of the ontology. The Human Aspect of Information Security Questionnaire was adopted as a comprehensive tool for gathering staff security practices as mediating variables in the framework.

**Conclusions:**

Data breaches occur often in health care today. This frequency has been attributed to the lack of experience of health care professionals in information security, the lack of development of conscious care security practices, and the lack of motivation to incentivize health care professionals. The frequent data breaches in health care threaten the mutual trust between health care professionals and patients, which implicitly impacts the quality of the health care service. The modeling and analysis of health care professionals’ security practices can be conducted with the PSC framework by combining methods of statistical survey, observations, and interviews in relation to PSC variables, such as perceptions (perceived benefits, perceived threats, and perceived barriers) or psychological traits, social factors, cultural factors, and social demographics.

## Introduction

### Background

Data breaches in health care are on the rise, emphasizing the need for a holistic approach to risk mitigation. According to IBM’s 2019 report [1], the cost of data breaches in the health care sector has remained the highest among all other sectors for the past 9 years. As of 2019, health care organizations registered the highest cost of data breaches (approximately US $6.5 million), which was 60% more than the cost reported by other industries [1]. Moreover, cyberattacks in health care are believed to represent a global phenomenon. In 2018, through the aid of a staff member, the health care records of about half the total population of Norway (3 million) were compromised [2]. The attack, which was considered as one of the biggest data breaches to have occurred in Norway, was described as a targeted method to access patient data at the Health South East Hospital. As a result, Norwegian citizens wondered whether health care data controllers were adopting reliable measures to secure the massive amount of sensitive health information collected from patients. In another incident, according to HealthCare IT News [3,4], a phishing attack compromised 38,000 patient records from Legacy Health based in Portland, Oregon in the United States. Personal data, such as patients’ email accounts, demographic information, dates of birth, health insurance data, billing details, medical data, social security numbers, and driver’s license information, were stolen. In a similar incident [3,4], about 1.5 million patient records, including data of the prime minister of Singapore, were breached. It was noted that the cybercriminals began by compromising front-end workstations, giving the attackers access to privileged user credentials. The attackers then escalated privileges to obtain access to the database. The breached data included demographic information, patient identification numbers, and medical information, such as diagnoses and test results. In the United States, about 365 breaches were reported in 2018, and hacking was the leading cause of health care data breaches, followed by other unauthorized access and disclosure incidents [5].

The use of information technology (IT) in health care (like in other sectors) has become indispensable [6]. Electronic health records now have multiple connections to health care professionals, patients, insurers, devices, and researchers [6]. The multiple points of access available to a larger number of stakeholders translates to multiple entry points and an increased attack surface. Additionally, health care professionals are usually busy with their core roles of restoring patients’ health, so little attention remains for focusing on information security [7,8]. Information security is instead often ignored to allow health care professionals to focus heavily on patients’ timely health restoration, especially in emergency care situations. This trade-off creates opportunities for adversaries to attack and gain access to health care systems [7,9-11].

Perimeter defenses have long been the default mechanism for providing information and network security and have therefore matured over the years. Perimeter defenses refer to securing the boundary between a company’s intranet and the public network (the internet) with physical security systems and technological countermeasures, such as firewalls, intrusion detection and prevention systems, security policy configurations, and antivirus systems [12]. Penetration through these perimeter measures is deemed more difficult and requires significant resources. Hackers therefore turn to explore easy entry points. With humans being the most vulnerable link in the security chain, attackers tend to exploit the human element to gain access to systems [13,14].

The health care context is characterized by high levels of trust between various social and peer groups [14-16]. This trust exists largely due to the identification of health care personnel through their professional training and socialization process [8]. Additionally, all health care practitioners typically value confidentiality as a result of the ethical principles and oaths, such as the Hippocratic Oath, that are core elements in health care professions [14-16]. This social and cultural bonding of health care professionals was identified as problematic for information security [14-16]. Health care professionals’ practices can also deliberately or inadvertently cause internal security breaches [3,14-17]. Furthermore, health care professionals have subtle variant behaviors in the usage of information communication technology in health care, which can threaten the confidentiality, integrity, and availability of personal health information [15,18,19]. The model of confidentiality, integrity, and availability is an information security model, which was developed to provide guidance for developing security policies to meet the availability, integrity, and confidentiality requirements of the assets of organizations [15,18,19]. Various researchers found that two-thirds of employees have contributed to data breaches [14-16,20] through mistakes or deliberate actions.

Security issues in health care have serious consequences [7,21,22]. Besides the potential loss of dignity, patients’ suffering may range from fraud to patient injury or death in health care–related data breaches [4,8,23,24]. Hospitals also experience a loss of trust and confidence from patients and other users if they experience data breaches. When hospital operations are interrupted, the cost of recovery from breaches is very high, especially in hacking related to ransomware [25,26]. Health care organizations can also face stringent sanctions from regulatory bodies, such as the General Data Protection Regulation (GDPR), or as a result of violating the Health Insurance Portability and Accountability Act (HIPAA) [24,27]. Violations of privacy and security regulations, such as the GDPR, by organizations in Europe could result in fines up to 4% of their annual global turnover or 20 million euros [28]. According to the International Organization for Standardization (ISO), the annual estimated losses from cybercrime could reach US $2 trillion in the near future, with countless daily additions of new breaches [29].

To this end, there is a need to assess the security practices of the human element in order to control data breaches in health care. Good security practices have been defined in regulations, policies, standards, guidelines, and codes of conduct, which are required to be implemented with both technical and nontechnical measures. However, to what extent do users comply with the established security policies? What are the challenges often faced by health care workers in their effort to comply with the prescribed security practices while doing their work? Are these security measures in conflict with the health care professionals’ health-related practices? How can the security requirements be improved for effective compliance while improving security effectiveness? How can health care workers be incentivized to better comply with security requirements while conducting their primary work? To protect the very sensitive nature of health care data, the health care domain needs to be properly modeled, assessed, and analyzed from the perspective of all possible entry points to mitigate attacks that are often associated with the psychological, social, cultural, and demographic characteristics of system users [30]. We therefore developed a comprehensive framework to uncover security issues caused by the human element termed in this paper as “health care professionals’ security practices.” This paper has been organized as follows. The *Theoretical Background* section provides details of the project, theories, and security practices used in the study, while the *Methods* section describes our adopted method. This is followed by a presentation of the results, followed by discussion of the results.

### Theoretical Background: Psychosociocultural Context

Amid the increasing frequency of data breaches in health care, all possible methods that can be used to model and analyze health care professionals’ security activities for security metrics should be considered. To this end, the Healthcare Security Practice Analysis, Modeling, and Incentivization (HSPAMI) project was introduced to model and analyze the security practices of health care professionals with the objective of assessing the gap between required security practices and current health care security practices [12]. The findings will support the development of solutions or incentives to improve health care professionals’ security behaviors.

The security practices of health care professionals are influenced by their personal characteristics, such as social demographics, perceptions, and other social and cultural factors. Psychological theories have been used in studies focusing on human behavior where the results could predict human information security practices [31]. Individual health care professionals’ security-related behavior can also be linked to their unique activities for constructing unique profiles in access control–related logs, such as browser histories, access logs, and network and operating system logs, in the context of big data [32]. Attack and defense simulations can also reveal health care professionals’ security behavioral risk levels. In using health care information systems, employees’ practices, induced by their characteristics, can have a positive or negative impact on information security [33]. Password management, physical security measures, users’ responses to phishing attacks, and users’ handling of resources entrusted to them by virtue of their user credentials are all examples of employee security practices [4]. The psychosociocultural (PSC) framework discussed in this paper focuses on perception and social, cultural, and sociodemographic variables. Therefore, the PSC framework depends on human behavioral theories, and individual- and work-related demographics [13] for assessing behavioral gaps in health care professionals’ security practices. Information security issues in health care can no longer be mitigated by technological countermeasures alone because the problem stems from health care professionals’ security practices, so enhancing “human firewalls” is necessary to mitigate the problem [11]. A human firewall involves strengthening the conscious security behaviors of health care workers in order to avoid security malpractices, such as falling victim to social engineering tricks. Strengthening the conscious security behaviors would augment the technological countermeasures, which would then enhance the overall security situation in health care. Frameworks for modeling and analyzing users’ security practices require comprehensive behavioral theories to study health care professionals’ practices for related security metrics and to identify potential mitigation strategies. Significant information security issues relating to psychological, sociocultural, and demographic factors could undermine information security policies and regulations, which could lead to information security violations [15].

PSC characteristics in this study refer to personal aspects, such as perceptions, attitudes, norms, and beliefs, as well as social and cultural factors that can influence the security practice of health care professionals [23]. Sociodemographic characteristics in this study include age, gender, education, workload level, work emergency situation, and security experience, while psychological, social, and cultural characteristics as a whole refer to health professionals’ security behaviors that are influenced by their psychological, social, and cultural factors, such as perceptions, workplace peer pressure, attitudes, norms, social bonding, and beliefs [23].

In a security practice analysis, the identified theories are usually related with various security practices. Peasons et al identified internet use, email use, social media use, password management, incident reporting, information handling, and mobile computing as comprehensive security practices in their survey work [34,35]. These security practices encompass a comprehensive list of the security practices that are most prone to security violations and compliance, and represent all sections of an information security policy that are essential to safeguard the confidentiality, integrity, and availability of information [4,35]. These security practices were compiled from the Human Aspect of Information Security Questionnaire (HAIS-Q) and from security standards and policies [35]. Other security practices were identified in previous studies [8,36], but the security practices in these studies were less comprehensive as compared to the HAIS-Q. Prior to usage, the HAIS-Q must always be updated to reflect current information security standards and policies [37].

### Security Practices

**Figure 1:**
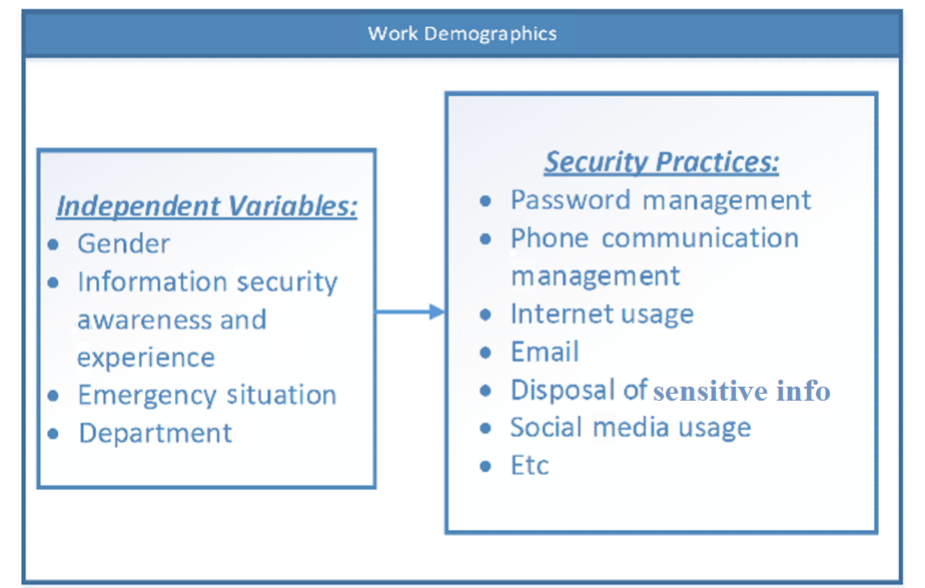
Relating independent variables with security practices.

As outlined in the HAIS-Q, health care professionals’ security practices include the security measures being adopted in the information security usage activities in response to security policies to safeguard the confidentiality, integrity, and availability of health care information systems. The requirements for such practices are usually expressed in regulations, directives, legislations, and security policies and specified in standards, best practices, and codes of conduct. Health care professionals’ security practices include security measures being adopted in the usage of the internet, email, and social media; password management; incident reporting; information handling; and mobile computing [24], as required by information security policies and standards. For instance, in password management, how do users respond to periodic password changes as required by some security policies? When modeling human behavior with these theories, independent variables (eg, professionals’ associated characteristics or constructs shown in Table 1 [4,8,14-16,18,21,34,35,38-40] and Figure 1) are often explored with mediating variables (Figure 1), such as the professionals’ security practices [25,26]. Therefore, comprehensive security practices are needed to address those aspects most prone to security violations, to ensure compliance, and to represent all sections of an information security policy that are essential for safeguarding the confidentiality, integrity, and availability of health care resources [27].

**Table 1:** Psychological, sociocultural, and demographic constructs.

Construct	Definition, hypothesis, and the effect on security practice
Social demographics	Social demographics refer to professionals’ demographics and work-related factors that influence their security practices [18]. Gender, workload, work emergency, role, department, and awareness or experience in information security all influence professionals’ security practices. During health care emergencies or some health care scenarios, health care professionals behave contrary to established security policies if the security measures obstruct health care or threaten patient privacy. Such behaviors adversely impact security [8]. Individual differences also influence security practices [38].
Psychological characteristics	Psychological characteristics in this study refer to an individual’s traits, perceptions, beliefs, thought processes, etc. These characteristics are influenced by various factors, including environmental factors [21]. Perceived threat severity, perceived susceptibility, perceived barriers, perceived self-efficacy, cues to action, attitude or personality, and emotions are some of the psychological characteristics that influence health care professionals’ security practices. If health care professionals increase their awareness of the adverse impact on security, they tend to behave more consciously [14,38].
Social factors	Social factors refer to the influence of peers and other professional groups. Social bonding, peer pressure, and trust level impact health care professionals’ security practices [4,21]. Due to trust and social bonding among health care professionals, conscious care behaviors tend to be adversely affected among them [15,16].
Cultural characteristics	Environmental norms, cultural beliefs, and assumptions impact security practices [4,21]. This study mainly focuses on organizational culture and excludes the potential effect of national cultures. However individuals’ cultural backgrounds also impact security-related behavior [34,35,39,40].

### Related Frameworks

In contributing to security conscious care behavior among health care workers, Humaidi et al developed a conceptual framework for determining the statistical significance of perceptions [31]. The study focused on security awareness and security technology related to health care professionals’ security conscious behaviors. Protection motivation theory (PMT) and health belief model attributes were used as independent variables to determine their impact on security awareness and security technology mediating variables.

Similarly, Cannoy et al employed the technology acceptance model (TAM), the theory of reasoned action (TRA), information assurance and security ethical behavior, organizational culture, and health information management [7] to develop a related framework. In the same context, Fernandez-Aleman et al advocated for more security awareness training to enhance good security practices and called for preventive and corrective actions to curtail incidents attributed to health care professionals [41]. The researchers studied the PSC context and some social demographic characteristics (age, gender, and experience). The security practices included password management, unauthorized access, disposal of sensitive information, and incidence reporting. The findings of the research provided some knowledge on the security gap between health care professionals’ required and actual information security practices.

Furthermore, the PMT and theory of planned behavior (TPB) [14] were adopted in a study to determine whether information security awareness, information security policy, and experience ultimately impact employee security practices. TPB relies on attitudes, subjective norms, and perceived behaviors to predict human behavior [42,43]. The PMT deals with the ability to protect oneself from threats based on the perceived severity of a threat, perceived probability of occurrence or vulnerability, impact of the recommended preventive practices, and perceived self-efficacy [14]. Additionally, Hassan et al proposed a conceptual model for determining the drivers of information security culture in the health care context [44]. Secondary data were explored for the framework, and the researchers proposed that information security culture is influenced by behavioral change management, information security awareness, security requirements, and organizational systems and knowledge.

Relatedly, Box et al reviewed the literature and proposed a model for information security compliant security practices within health care environments [16]. The researchers aimed to provide an overview of factors that were influencing or discouraging information security compliance. The constructs used in the model included compliance-promoting and misuse-deterrence factors, body of knowledge, attitudes, skills, behavioral interventions, and security compliant behavior.

In an effort to improve health care professionals’ conscious care behavior, van Deursen et al aimed to understand the sociotechnical risks of information security in the health care sector [45]. The study excluded the technical aspects of information security risks but focused on information security risks related to human and organizational factors. The researchers explored security incidents recorded in a central database by the Freedom of Information officers of the Scottish Health Boards and English Care Trusts.

Various theories are used to model and assess the security practices of users. Cheng et al identified such theories, including the TRA/TPB, general deterrence theory, PMT, and TAM, as the most widely used theories for studying human security practices in the PSC context [33]. The systematic review provided knowledge in common theories, but guidelines were not provided on the selection and application of these theories.

Similarly, Yeng et al surveyed for related theories, security practices, and evaluation methods [4]. They found various theories that can be employed in modeling and analyzing health care security practices, as shown in Multimedia Appendix 1; however, the approach was less systematic and lacked a framework.

Health care security practices are not only impacted by social demographic traits (eg, age, gender, and experience) [27,46,47] or psychological traits, but also potentially influenced by other critical factors, such as emergency situations and workload, as shown in Figure 1.

In view of the shortfall of the above framework to allow for the efficient study of health care professionals’ security practices, we proposed the PSC framework to create a holistic set of health care professionals’ characteristics for analyzing a wide range of security practices.

### Problem Specification, Scope, and Contribution of the Study

Information security issues attributed to the human element have been recognized to be as important as technological security measures. Therefore, various frameworks have been developed in the PSC context, but none is comprehensive within this study scope. Some of the frameworks were developed to assess only perception variables [4,26,33,36,37,40]. Other frameworks adopted only social constructs [4,7,35,42,43] or cultural factors [33,48,49]. However, in a scenario where a study must be conducted with the aim of comprehensively understanding and addressing the information security challenges often faced by health care professionals, it is important to know which of the existing frameworks will be adequate. The reviewed frameworks [8,14-16,31,38,41,44,45,49-66] were not fully comprehensive. Meanwhile, security issues are affected by all these aspects and not just psychological, social, cultural, or sociodemographic aspects alone [38]. Therefore, a framework that can include all these aspects (Multimedia Appendix 1) will be a comprehensive one. Furthermore, it is necessary to systematically structure the knowledge in a way that explicitly shows the connection between concepts in the study domain by using appropriate methods such as a domain ontology.

This study proposes a holistic framework that consists of psychological, sociodemographic, and sociocultural variables, which can be used to analyze a comprehensive set of health care professionals’ security practices, as shown in Table 1.

The framework builds on studies collected in a literature review, as shown in Multimedia Appendix 2. In order to comprehensively and explicitly represent the domain of interest, we also produced a domain ontology for developing the PSC framework. The purpose of the ontology is to enable the creation of a common understanding among people or software agents within a domain to share, reuse, and analyze domain knowledge [67,68]. The security issues in health care organizations not only are attributed to health care workers’ behaviors, but also stem from security awareness and organizational factors, such as IT competence of business managers, environment uncertainty, industry type, organizational preparedness, organizational culture, top management support, and organizational size. Various studies identified that organizational factors, including organizational size and industry type, have strong influences on IT [69-71] and implementation of information security management [72]. Notwithstanding, the scope of this study does not cover all organizational factors, but considers organizational factors and top management, with much focus on security issues directly involving health care workers, such as health care professionals who provide therapeutic measures (doctors, nurses, pharmacies, laboratory personnel, radiology officers, etc), IT personnel, health administrators, and finance personnel. The next section outlines the methods used in this study.

## Methods

### General Approach

We conducted a literature review of the state-of-the-art theories and security practices in health care in order to develop a holistic framework. According to previous reports [73-76], there are various types of systematic studies. These include systematic mapping studies and systematic literature reviews. Systematic mapping studies perform reviews of topics in a broader sense by categorizing basic research articles into specific areas of interest. Systematic mapping studies have general research questions aimed at determining research trends or state-of-the-art studies. Systematic literature reviews aim to aggregate evidence and therefore have a relatively specific research goal. To this end, a systematic mapping study was adopted in this work [73,74]. Based on a review, we built and used an ontology to develop the PSC framework, which covers most of the dimensions of health care professionals’ security-related traits. This framework allows for holistically analyzing health care security practices.

**Figure 2:**
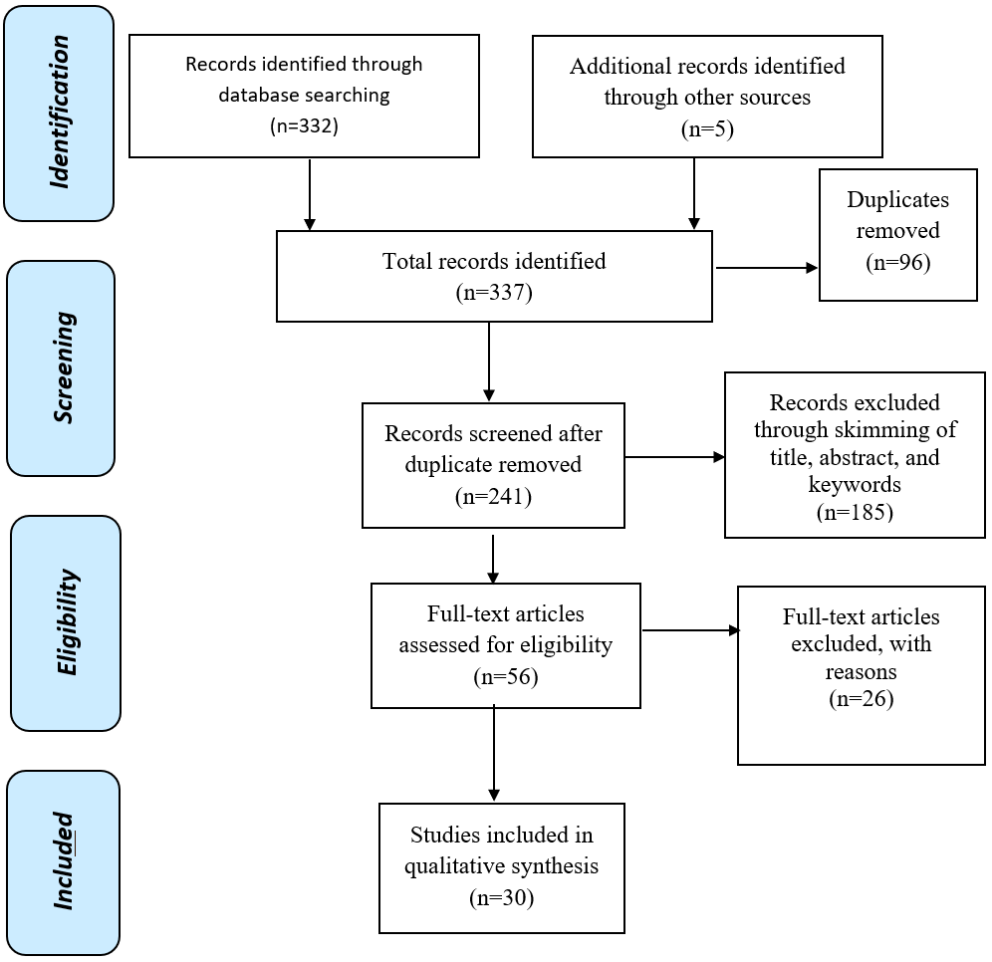
PRISMA (Preferred Reporting Items for Systematic Reviews and Meta-Analyses) flowchart.

The literature search was conducted between June 2019 and December 2019 through Google Scholar, Science Direct, Elsevier, IEEE Explore, ACM Digital, PubMed, and Scopus. Different keywords, such as “healthcare,” “health,” “staff,” “employee,” “professional,” “information security,” “behavior,” and “practice” were used. To ensure a good-quality search strategy, the keywords were combined using the Boolean functions “AND,” “OR,” and “NOT.” Peer-reviewed journals and articles were considered. The inclusion and exclusion criteria were developed based on the study objective and through discussions among the authors. Initially, 337 articles were selected by skimming through the titles and keywords for articles that aligned with the inclusion and exclusion criteria. Screening was further applied by quickly reading the abstracts and keywords. Duplicates were then filtered out, and articles that appeared relevant, based on the inclusion and exclusion criteria, were read in their entirety and evaluated. Twenty-six articles were further removed from the study in the full reading and evaluation stage based on various reasons, including limited scope and articles not meeting the inclusion and exclusion criteria. For instance, a study [77] looked into security issues in health care using a machine learning approach, but this was out of the scope of this study. Furthermore, another study [78] looked into an assessment model for software quality issues in health care, but security was not the main focus. Based on these and other similar reasons, the number of articles included in this study reduced greatly. Other relevant articles were also retrieved through the reference lists found in the literature. Figure 2 presents a Preferred Reporting Items for Systematic Reviews and Meta-Analysis (PRISMA) flow diagram that clarifies article selection and screening [79].

### Inclusion and Exclusion Criteria

Articles included in the review were required to be about security practices in the health care context and to pertain to health care professionals’ information security behaviors in relation to their work. Other articles, such as those that were not related to the health care context and did not focus on human behavior in health care, were excluded.

### Data Collection and Categorization

Data collection and categorization were established from the study objective through completion of the literature review and based on discussions of the authors. In order to assess, analyze, and evaluate the study, these categories were exclusively defined as follows:

1. Theory used: This category included only theories (psychological, social, or cultural theories) used in the study to relate human characteristics to security practices.
2. Security practice: This category included the security measures (eg, password management, incident reporting, and internet usage) used in the study.
3. Study type: This category specified the type of study, whether theoretical or empirical. In this study, “empirical” refers to practical studies conducted in the health care context and “theoretical” refers to reviews and proposed frameworks for related studies.
4. Study context: This category specified what area (eg, psychological, social, cultural, or demographic context) the study covered.

Multimedia Appendix 2 presents the categorization of the included literature.

### Literature Evaluation and Analysis

The selected articles were assessed, analyzed, and evaluated based on the above defined categories. We performed an analysis on each of the categories (theory used, security practice, study type, and study construct) to evaluate the state-of-the-art approaches. The percentages of the attributes for the categories were calculated based on the total number of counts (n) of each attribute type. Some studies used multiple categories; therefore, the number of counts for these categories exceeded the total number of articles in the study.

## Results

### Literature Review Findings

This section presents the findings of the literature review, the ontology, and the proposed theoretical framework.

The searches in the aforementioned online databases resulted in a total of 337 records being initially identified by following the guidelines of the inclusion and exclusion criteria in the reading of titles, abstracts, and keywords. We further screened and selected articles by reading the objective, methods, and conclusion sections of each study, and this led to a further exclusion of 185 articles that did not meet the defined inclusion criteria. A total of 96 duplicates were also removed, and the remaining 56 articles were fully read and appraised. After the full-text reading, a total of 30 articles were included and analyzed in the study (Figure 2).

Table 2 presents the theories identified in the literature review [4,7,11,14,49,53,59,62,65]. The theories that were most often used in analyzing the security practices of health care professionals included the health belief model (n=6), TPB (n=5), general deterrence theory (n=4), PMT (n=4), and technology acceptance theory (n=2), as shown in Table 2.

**Table 2:** Psychological, social, and cultural theories.

Theory	Count, n
Health belief model [49]	6
Theory of planned behavior [14]	5
General deterrence theory [53]	4
Protection motivation theory [14]	4
Technology acceptance theory [4]	2
Technology threat avoidance theory [59]	1
Social bond theory [11]	1
Situational crime prevention [53]	1
Institutional theory [62]	1
Grounded theory [65]	1
Social control [7]	1
The big five theory [7]	1

The security practices that were often related with the individual characteristics of the health care professionals at their workplaces included password management (n=6), unauthorized disclosure (n=3), security policy and procedures (n=3), and email use with sensitive data (n=2), as shown in Table 3 [4,41,45,50,51,60].

The categories of theories frequently identified included psychology (n=7), demographics (n=6), social (n=3), and cultural (n=3), as shown in Table 4.

**Table 3:** Security practices.

Security practice	Count, n
Password management [41,45,51]	6
Security policy and procedure [60]	3
Unauthorized discloser [60]	3
Email use with sensitive data [4]	2
Logging off session [4,50]	2
Emergency access [4]	2

**Table 4:** Categories of the studies identified.

Category	Count, n
Psychology	7
Demographics	6
Social	3
Cultural	3
Linguistics	1

A higher proportion of empirical studies (n=15) was identified, compared with theoretical studies (n=9).

### Proposed Ontology

**Figure 3:**
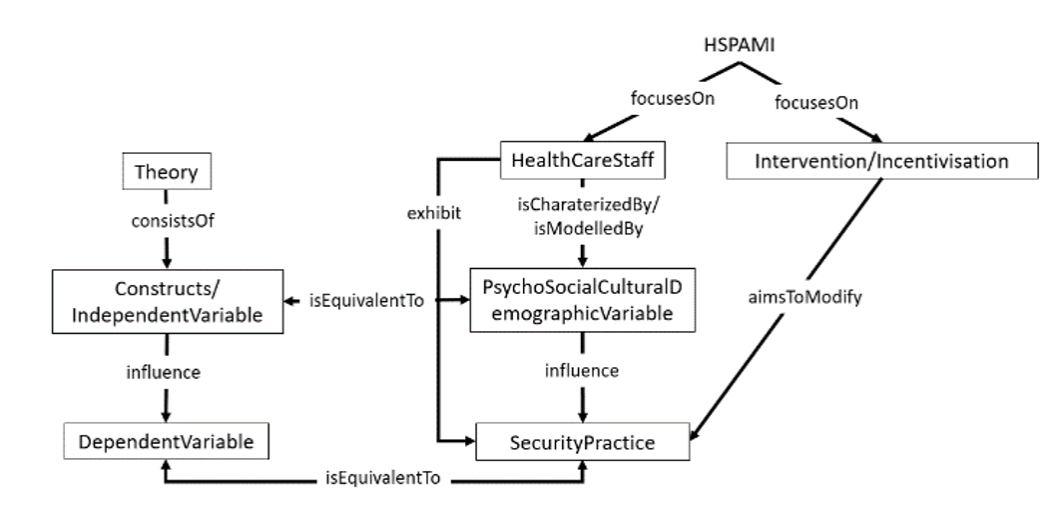
Structure of the ontology representing concepts as classes and specifying the relationship among the classes. The relationships among concepts are represented by the arrows between concepts in the rectangles. HSPAMI: Healthcare Security Practice Analysis, Modeling, and Incentivization.

Ontologies are formal specifications of key concepts within a domain and the relationships among them. Ontologies are purposeful artefacts that make domain assumptions explicit, enable the construction of a common understanding among stakeholders, enable the reuse of expert knowledge, etc [51]. The proposed ontology contained a total of eight distinct concepts and nine relationships, which enabled us to capture the conceptual relationship between a total of 76 unique instances extracted from the literature. Figure 3 presents the ontology capturing key concepts of the HSPAMI project and the supporting empirical evidence that corresponds to the PSC framework. The following subsections describe the steps followed for the construction of the ontology based on the guidelines presented in a previous report [67].

#### Development of the Ontology

The main objective of the proposed ontology was to map the HSPAMI main study areas to empirically supported research results in order to develop a literature-based comprehensive holistic framework that can be utilized in the project and by researchers or practitioners interested in the domain of information security within the health care context [4].

#### Determine the Domain and Scope of the Ontology

The proposed ontology aimed to (1) structure the main focus areas of the HSPAMI project, (2) create a connection between these study areas and existing empirical research results, and (3) develop a comprehensive PSC framework that efficiently communicates domain knowledge to various stakeholders. Thus, the domain is defined as health care professionals’ security practices, and the scope is restricted to research results investigating the relationship between psychological and sociocultural theories and variables with respect to security behaviors.

### Use of Existing Ontologies

Literature searches were conducted for existing comprehensive domain ontologies on Google Scholar, ScienceDirect, and Scopus, with the following keywords: “ontology,” “healthcare,” “security behavior,” and “practice.” These keywords were also combined with the Boolean functions of “AND,” “OR,” and “NOT.” No comprehensive ontology was identified. Ontologies that explicitly model and structure the domain have been proposed for various purposes in the health care domain, such as interoperability [80] and regulating access control for internet of things–based health care [40,81]. The ontology proposed in this paper uses the HSPAMI study areas as an organizing principle for the existing empirically supported research results [40,81].

#### List of the Relevant Terms of the Domain

The fundamental concepts were identified in a previous report [4] with respect to the main study areas of the HSPAMI project. These are health care professionals’ psychosocial and cultural demographic variables, security practices, and incentivization of security practices. The concepts were aligned with the classes commonly encountered in empirical studies investigating the relationship between theoretical constructs and behaviors of interest or outcome variables (eg, security practices).

#### Define the Classes and the Class Hierarchy

In order to represent the relationship between concepts of the domain and empirical research results, the classes were conceptually connected to each other. The combination approach was followed in defining the classes and hierarchy, which combined top-down and bottom-up approaches. More salient concepts (HSPAMI concepts and study components) were defined first, and then, based on the identified empirical results, more specific concepts were included. To deal with different terminologies applied to similar concepts (synonyms), the equivalence of classes was represented by the “isEquivalentTo” relationship between concepts, which was inherited by the instances added to the classes. Thus, theories that consisted of constructs could be included in the ontology by defining and connecting an instance to the accompanying theory. Variables that were not specifically part of any theory (eg, demographic variables) could be included by restricting the domain attribute to the class of constructs. Table 5 shows the existing classes defined within the ontology, with example instances. Based on the literature review, a total of eight classes were defined as the most general concepts, as shown in Figure 3.

**Table 5:** Main concepts defined as classes.

Classes	Instances
HSPAMI^a^	—^b^
HealthCareStaff	Doctors, nurses, etc
Intervention/Incentivization	Motivation, deterrence, etc
PsychoSocialCulturalDemographicVariable	Gender, age, etc
SecurityPractice	PasswordManagement, EmailUse, etc
Theory	Theory of planned behavior, protection motivation theory, etc
Construct/IndependentVariable	Attitude, SubjectiveNorm, etc
DependentVariable	ActualBehavior, SecuriyAwareness, etc

^a^HSPAMI: Healthcare Security Practice Analysis Modeling and Incentivization.

^b^No instance.

#### Define Properties of Classes

The main objective of this step was to describe the relationship of a class to other individuals. The properties were defined at the most general class; thus, all members of that class inherited the given property. Table 6 shows the relationships and the connected classes in the proposed ontology. A total of nine properties link various concepts in the ontology.

**Table 6:** Relation of classes.

Relation of classes	Classes connected
consistsOf	Theory - Construct
influence	IndependentVariable - DependentVariable
isEquivalentTo	Construct - PsychoSocialCulturalDemographicVariable
exhibit	HealthCareStaff - SecurityPractice, DependentVariable
isCharacterizedBy/isModeledBy	HealthCareStaff - Construct
aimsToModify	Intervention/Incentivization - SecurityPractice
focusesOn	HSPAMI^a^ - Intervention, HealthCareStaff
isATypeOf	Gender - Construct
hasAttribute	SelfEfficacy - Psychological; Gender - Demographic

^a^HSPAMI: Healthcare Security Practice Analysis Modeling and Incentivization.

### Define the Data-Type Properties

This step was excluded in the development of the ontology at this stage. Since ontologies can be developed at various levels of granularity, these steps may be iteratively completed at a future stage when the requirements (eg, development of software) are defined more specifically. For the purpose of creating a comprehensive framework of health care staff characteristics and security practices, this step was unnecessary.

#### Create Instances

The research papers meeting the inclusion criteria were subsequently analyzed in detail to extract instances for the previously enumerated classes. The list of papers reviewed for constructing the ontology are presented in Multimedia Appendix 3.

**Figure 4:**
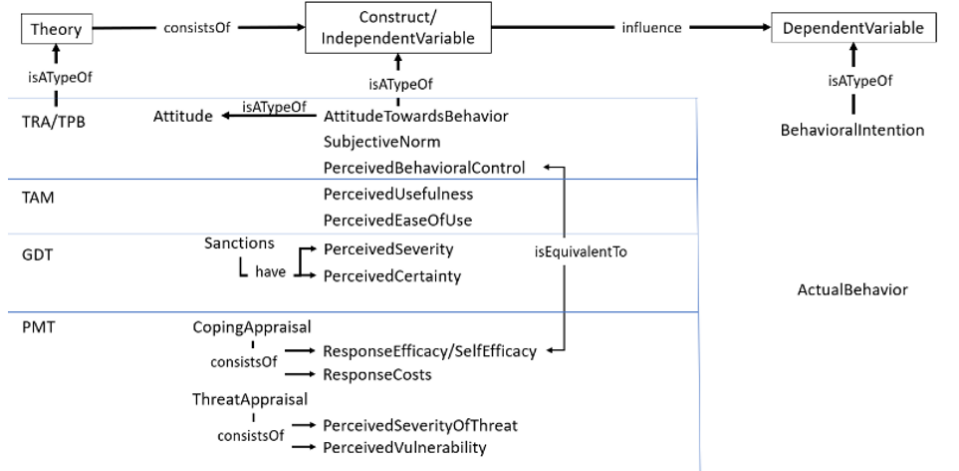
Instances and additional properties defined from the review paper [38]. GDT: general deterrence theory; PMT: protection motivation theory; TAM: technology acceptance model; TPB: theory of planned behavior; TRA: theory of reasoned action.

**Figure 5:**
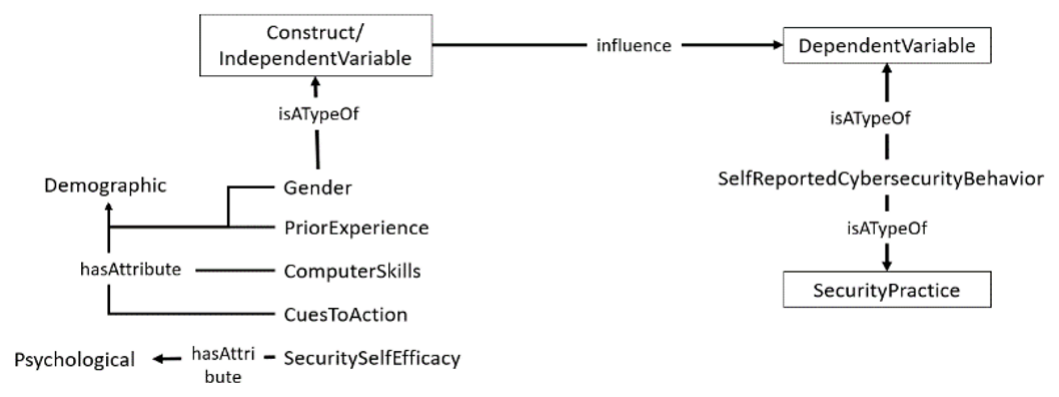
Expansion of the ontology based on results from a previous report [38].

For the purpose of demonstration, Figure 4 and Figure 5 present how instances can be included in the existing ontology. Additional properties (eg, equivalence of classes) can be represented, which is especially important to avoid ambiguity and for clarifying the semantic meaning of different concepts when they are related (eg, self-efficacy is equivalent to perceived behavioral control). Each theory discussed in a previous report [82] was represented as an instance of the theory class, and the object property “isATypeOf” was proposed to capture the relationship. The TPB consisted of the following three constructs: “AttitudeTowardBehavior,” “SubjectiveNorm,” and “PerceivedBehavioralControl,” which can be considered equivalent to beliefs related to self-efficacy.

### Ontology and the PSC Framework

**Figure 6:**
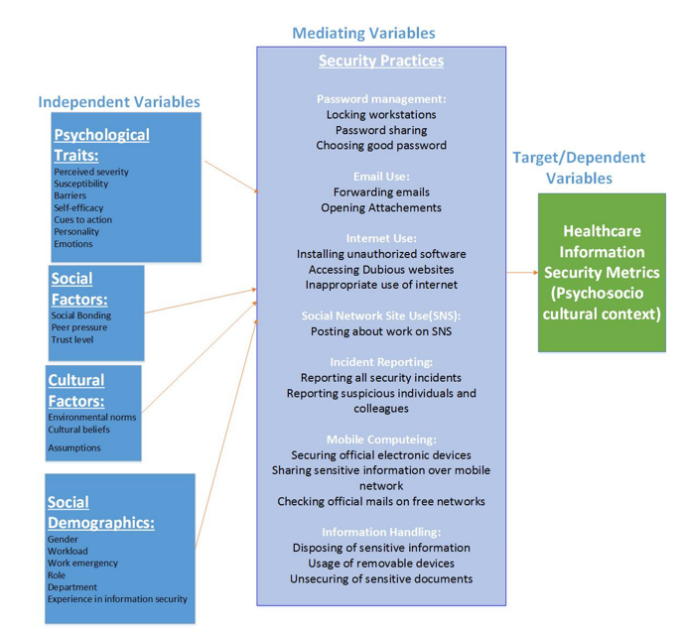
Proposed psychosociocultural framework.

The framework shown in Figure 6 consists of independent variables, mediating variables, and the dependent or target variable. The independent variables have various constructs, including psychological traits, social factors, cultural influences, and sociodemographic characteristics. Attributes of these constructs were associated with comprehensive security practices. The security practices served as mediating variables. The target or dependent variable, known as health care professionals’ security metrics, was obtained after relating the independent and mediating variables. The framework components are as follows:

1. Independent variables: This aspect of the PSC framework consists of the characteristics of the health care staff that can impact health care professionals’ security practices. With reference to Figure 4 and Figure 6, these characteristics are segregated into psychological or perception variables, sociodemographics, and social and cultural attributes. The psychological traits include perception variables or constructs, such as perceived severity, perceived susceptibility, perceived cues to action, perceived barriers, and perceived self-efficacy, personality, and emotions.
2. Social bonding: Social bonding is related to social behaviors that can influence health care professionals’ information security behaviors. Such constructs include social bonding, peer pressure, and trust level, as shown in Figure 6.
3. Cultural factors: Culture-related traits that can impact information security include environmental norms, beliefs, and assumptions.
4. Social demographics: Social demographics, such as gender, workload, information security experience, emergency, role, and experience, are hypothesized to have an impact on information security relating to health care staff.

Figure 4 presents the expansion of the ontology with empirical results that have particular theories associated with them. Psychological, cultural, and demographic variables were grouped by defining additional attributes to facilitate knowledge sharing.

The PSC framework also has mediating variables that are basically the security practices of the health care staff. The health care security practices are the required security-related behaviors defined in the policies, standards, regulations, and codes of conduct for health care personnel. Health care staff are therefore required to abide by such security measures to enhance the confidentiality, integrity, and availability of health care data. The security practices in the PSC framework were adopted from the HAIS-Q. The HAIS-Q is a framework consisting of a comprehensive information security practice. In a typical health care environment, health care staff members go through their daily security practices within the scope of the HAIS-Q, and these security practices are impacted by independent variables. Security practices include social network usage, password management, incident reporting, mobile computing, and internet use, as shown in Figure 6.

Finally, the target or the dependent variable is the measured security practice of health care staff. Such a security metric can therefore be used for management decision-making, such as implementing intervention measures aimed to improve conscious care security practices.

## Discussion

### Principal Findings

Information security management for mitigating data breaches involves identifying the threats to information security and devising efficient countermeasures [28]. Information security management includes adding tools and serving employees with checklists of information security user policies for work roles, as well as requiring employees to abide by those policies. However, the security of health care data also requires systematic analysis of the health care professionals’ security practices for building a “human firewall,” with the objective of enhancing a conscious care and security resilience culture. Thus, identification of various sources of human threats in the social, cultural, and psychological contexts is vital [12,34,35,39].

To this end, we identified constructs capturing psychological, sociocultural, and demographic variables (termed in this study as “psychosociocultural context”) to develop the PSC framework to understand health care professionals’ security practices. The main contribution of this paper is the development of the PSC framework implemented as a domain ontology. Specifically, the framework includes concepts and important variables that have been empirically proven to influence the behavior (ie, security-related practices) of health care professionals when dealing with sensitive information in a health care work setting.

Based on the overview of existing literature [8,14-16,31,38,41,44,45,49-66], we concluded that existing frameworks lack a comprehensive and holistic perspective. Furthermore, not all frameworks provide strong empirical support for the inclusion of variables from the perspective of both security related-behaviors and professionals’ characteristics [14,45,49,52,55,57-59]. Therefore, this paper represents a step toward creating a comprehensive and practically useful framework that can aid information security practitioners in fulfilling their work requirements by incorporating relevant concepts and research results that serve as a foundation of the framework.

The utility of the proposed framework will be tested in the HSPAMI project by scoping the forthcoming investigations on factors that must be considered in monitoring and modifying health care professionals’ security-related behaviors. While specific empirical research papers are necessarily limited with respect to their scope on the security practices and the theories utilized, such papers provide the crucial building blocks of the overarching framework. The first major advantage of the present framework is that it encompasses accumulated knowledge by utilizing the evidence from previous investigations (each focusing on narrowly defined behaviors [8,33,35,38,45,48,82-84], eg, responding to spam and sharing information on social media); thus, the framework provides a more comprehensive perspective on the various forms of security-related behaviors that should be investigated. This aspect of the present framework is mainly supported by the inclusion of the concepts found in the HAIS-Q instrument, which is a validated and widely utilized questionnaire for measuring information security–related beliefs, knowledge, and attitudes [34,35,39].

Based on the literature survey, we also developed an ontology to include significant concepts for the development of the PSC framework. Within the PSC context of health care professionals’ security practices, various studies exist [14,31,41]. The second major contribution therefore involves the selection of psychological, social, and demographic variables (ie, constructs and theories) from existing literature [8,33,35,38,45,48,82-84] and the representation of the framework in the form of a domain ontology. By specifying the framework as an ontology, we can efficiently structure, organize, and reuse the vast amount of existing knowledge. Furthermore, the ontology also enables an efficient way to share information with other stakeholders within and outside the HSPAMI project without ambiguities, thus helping to build a common understanding. This aspect is exemplified by object relations that link synonyms or different terminologies used for the same construct to build a common language shared by all stakeholders involved in project-related activities. Finally, the ontology may as well serve as a blueprint for applications developed within the project, such as relational databases containing relevant variables and specifying the connections between them.

Evaluation of the ontology refers to judgments about the technical features of the ontology and assessment of its usability and utility. Generally, evaluation aims at ensuring the correctness and completeness of an ontology [85]. It is an iterative process, which can be conducted at each point of the ontology’s life cycle. An evaluation must be done against a frame of reference, which may be a set of competency questions and requirements, and the real world [85], and may take the form of a technical evaluation in the lab or at the location of application (eg, health care context with health care professionals). Evaluation may be performed with several criteria as follows: evaluation of definitions (checking for the absence of well-defined properties in the ontology), structure of the ontology (matching the ontology’s structure with the design criteria of the environment, where it is intended to be used), syntax of definitions (ensuring that syntactically correct keywords are present), content of definitions (identifying what concepts are covered and what concepts are not included or included incorrectly), consistency (avoiding contradictions), completeness (extent of covered concepts in the domain of interest), and conciseness (checking whether information contained in the ontology is relevant and accurate) [85]. As the ontology has been developed using existing empirical research results, its validity partially depends on the reliability and validity of the findings in the knowledge base. Furthermore, at this stage of development, only a technical evaluation is possible; thus, its validation in real-world settings is among the key goals of future work. Eventually, the practical benefits of the ontology depend on its recognition and approval among experts who utilize it [86].

With respect to the comprehensiveness of the current PSC framework, it is comparable to similar approaches [7,31] with a stronger focus on the requirement that only empirically supported research results are included. While this may limit the comprehensiveness of the framework, it ensures that only relevant and practically significant theories and concepts are investigated and applied during the activities of the overall project, which can save time and other valuable resources during the process. The real-world evaluation of the framework in terms of its usefulness for sharing and analyzing knowledge, creating a common understanding, and representing concrete aspects of the envisaged application domain will be studied within the scope of the project through case studies, field experiments, or other research methods.

To complement the efforts of health care professionals in maintaining the confidentiality, integrity, and availability of health care data, a systematic approach to identify the detailed and subtle health care professionals’ characteristics that impact information security practices must be applied. All these constructs are vital when measuring the conscious care behavior of health care professionals. For example, if we assume that psychological constructs are not measured in a typical empirical study of security conscious care behaviors, there will be a gap since the perception of the health care security practice will not be captured [12]. Thus, if security solutions are professed based on such a study, the solutions will lack measures to deal with the perception aspect.

Therefore, through the PSC framework developed in this paper, we identified various constructs within the project domain. The holistic approach is much needed because it strives to capture the entire problem area in the scope of the project. Focusing on just one or two aspects of staff-related traits that impact security in the health care industry might not be sufficiently effective [12]. For instance, some of the frameworks focused only on social factors, with the exclusion of other factors, such as the perception. Without determining how health care staff perceived the severity of the impact of their information security malpractices in a related study, health care professionals may not be treated with appropriate incentivization methods for improving such malpractices. Lack of perception variables implies that health care staff would not be able to perceive the gravity of their security-related malpractices, which means there may still be data breaches resulting from untreated psychological traits. Conversely, if a study is conducted with only psychological constructs, data breaches may still occur as a result of untreated social-related constructs, such as social bonding and peer pressure. An approach, such as the PSC framework, therefore appears necessary for an efficient study.

### Conclusion and Future Work

The mutual trust between health care professionals and their patients is under threat owing to frequent and large data breaches in health care. Furthermore, the richness of health care data is attracting cyber criminals. Since scaling universal technological security measures is challenging, cyber criminals tend to exploit health care staff for easy entry.

To curtail this ascendance in data breaches, a comprehensive set of health care professionals’ characteristics and security practices, which can impact information security, was identified. An ontology was developed from the identified literature generated by a literature review. Then, a holistic PSC framework was developed. The framework can be implemented with a mixed method approach encompassing both qualitative and quantitative studies [45,87].

Owing to the systematic approach used to develop the PSC framework, it is possible to identify reliable security metrics while considering all the subtle characteristics of health care professionals and their related security practices. Such metrics can then be used to develop incentivization or motivational measures aimed toward building stronger “human firewalls” to curtail data breaches in health care. Beyond the conventional qualitative evaluation methods of interviews and questionnaires or surveys, other approaches, including team-based learning [87] and the Delphi method [45], should be explored in the future to enrich empirical studies using comprehensive frameworks such as our PSC framework. Additionally, organizational factors should be considered in the future, since they were not entirely covered in this study.

Furthermore, clarifying the meaning and interconnectedness of various terms imported from different domains (eg, psychology, information security, sociology, etc) can be beneficial for discovering contradictory or converging pieces of evidence revealed by researchers. While the ontology currently captures only a limited number of concepts from the PSC and demographic contexts of health care professionals, it is flexible and can be extended with new results based on advances in the literature. The level of granularity can, for instance, be increased depending on the requirements of the applications in future work. The emphasis on empirical foundations could also be strengthened by representing associations between variables through specifying additional object properties associated with the classes (eg, correlations, predictive accuracy, etc). The compatibility of this domain ontology with other ontologies (eg, health care staff demographic characteristics in employee databases) needs to be investigated in future work to increase reusability and to achieve more realistic mapping between research results and the opportunities to observe the variables included in the framework. Additional expert knowledge could be useful for enriching the framework, and this can be achieved through iterative workshop sessions with other stakeholders (eg, health care staff, security practitioners, etc).

## Appendix

Multimedia Appendix 1 Analysis of the theories and their application areas in the Healthcare Security Practice Analysis Modeling and Incentivization (HSPAMI) project [<xref ref-type="bibr" rid="ref4">4</xref>].

Multimedia Appendix 2 Summary of the literature review.

Multimedia Appendix 3 Articles used to construct the ontology.

## Abbreviations

GDPR: General Data Protection Regulation
HAIS-Q: Human Aspect of Information Security Questionnaire
HSPAMI: Healthcare Security Practice Analysis Modeling and Incentivization
IT: information technology
PMT: protection motivation theory
PSC: psychosociocultural
TAM: technology acceptance model
TPB: theory of planned behavior
TRA: theory of reasoned action
