# Supplementary material for: Mapping the Psychosocialcultural Aspects of Healthcare Professionals’ Information Security Practices: Systematic Mapping Study
Source: JMIR Hum Factors. 2021 Jun 9;8(2):e17604. doi: 10.2196/17604 (PMC8235336; doi:10.2196/17604)
Supplement: Multimedia Appendix 1 [file humanfactors_v8i2e17604_app1.docx]

**Multimedia Appendix 1.** Analysis of the theories and their application areas in the Healthcare Security Practice Analysis Modeling and Incentivization (HSPAMI) project [4].

| HSPAMI Study Area | Theories That  Support Study Area |
| --- | --- |
| Social bonding | Social Control [34] |
| Peer pressure | Social Control [34] |
| Social norms and beliefs | SC [8], Health belief model (HBM)[34, 49, 50] |
| Healthcare emergency | Protection motivation theory (PMT), Social Control, Theory of planned behavior (TPB)[4, 15, 16, 34, 35, 81] |
| Workload | PMT [4],[15, 16, 35] HBM [49, 50, 81] |
| Privacy and security perception | PMT, Deterrence theory [4], HBM [34, 49, 50, 81] |
| Personality and attitude | The big five model (TBF), TPB, HBM [15, 40, 49] |
| IS experience, education, and knowledge | PMT [49] |
| Emotions | PMT [50], HBM [15, 40] |
