# Supplementary material for: Mapping the Psychosocialcultural Aspects of Healthcare Professionals’ Information Security Practices: Systematic Mapping Study
Source: JMIR Hum Factors. 2021 Jun 9;8(2):e17604. doi: 10.2196/17604 (PMC8235336; doi:10.2196/17604)
Supplement: Multimedia Appendix 2 [file humanfactors_v8i2e17604_app2.docx]

**Multimedia Appendix 2.** Summary of the literature review.

|  | Ref. | Theory Used | Security Practice | Context Used | Study Type |
| --- | --- | --- | --- | --- | --- |
| 1 | [15] | The protection motivation theory and theory of planned behavior (TPB) | Information security awareness, security policies and procedures, security experience | Psychological, demographic | E |
| 2 | [50] | Health belief model (HBM) | Security, awareness user’s health, information system’s security compliance behavior | Psychological, demographic | E |
| 3 | [60] | Technology threats avoidance theory |  | Psychological, demographic (gender, age, and position) | E |
| 4 | [59] | Organizational culture characteristics and health belief model (HBM) |  | Culture | E |
| 5 | [58] |  | Security education, security technology investment |  | E |
| 6 | [57] |  |  | Culture | T |
| 7 | [16] | Literature review, coping model of user adaptation and framework for classifying emotions as a tool |  |  | T |
| 8 | [17] | Literature review |  |  | T |
| 9 | [46] | Delphi study | Staff leaving data assets unattended on the premises, and these assets consequently go missing; staff sharing passwords to access patient data; and staff sending email containing personal patient data to the wrong addressee, thus disclosing data to unauthorized persons |  | E |
| 10 | [56] | General deterrence theory | Discloser of sensitive information | Social | E |
| 11 | [55] |  | Adherence to organizational policy |  | T |
| 12 | [54] | General deterrence theory, social bond theory , social learning theory , theory of planned behavior (TPB), situational crime prevention |  | Social | T |
| 13 | [53] | Health belief model (HBM), theory of planned behavior (TPB), and the health action process approach |  | Psychological | E |
| 14 | [52] |  |  | Demographic | T |
| 15 | [51] |  | Password management, logging off sessions |  | T |
| 16 | [61] |  | Security awareness, security policy, organizational culture, security culture |  | T |
| 17 | [32] | Protection motivation theory (PMT) and health belief model (HBM) |  | Psychological | T |
| 18 | [62] |  | Password management, unauthorized access |  | E |
| 19 | [63] | The institutional theory | Security policy |  | T |
| 20 | [64] |  | Self-efficacy to comply, patient's medical status |  | E |
| 21 | [42] |  | Password management, confidential information discarding, unauthorized access via computer screen, reporting security violations |  | E |
| 22 | [65] | Vocabulary test |  | Linguistic | E |
| 23 | [45] |  | Information security awareness, knowledge |  | T |
| 24 | [9] |  | Authentication, de-authentication, permission management |  | E |
| 25 | [66] | Grounded theory (GT) | Access control, access control policies, access in emergency situations, access control solutions |  | T |
| 26 | [67] |  | Authentication |  | E |
| 27 | [35] | Health belief model (HBM) (Rosenstock, 1974) and protection motivation theory | Computer skills, experience with cyber security practice | Demographic, Psychological | E |
| 28 | [8] | Technology acceptance model (TAM) |  | Psychological | E |
| 29 | [84] | Theory of planned behavior (TPB), general deterrence theory |  |  | T |
| 30 | [4] | Health belief model (HBM), protection motivation theory (PMT), theory of planned behavior (TPB), the big five (TBF) model, and social control | Internet use, email use, social media use, password management, incident reporting, information handling, and mobile computing | Psychological, social, cultural, demographics | T |

E= “empirical study”

T= theoretical study”
