# Supplementary material for: Mapping the Psychosocialcultural Aspects of Healthcare Professionals’ Information Security Practices: Systematic Mapping Study
Source: JMIR Hum Factors. 2021 Jun 9;8(2):e17604. doi: 10.2196/17604 (PMC8235336; doi:10.2196/17604)
Supplement: Multimedia Appendix 3 [file humanfactors_v8i2e17604_app3.docx]

| Instances extracted from empirical studies | Classes containing instances | Sources |
| --- | --- | --- |
| TRA/TPB | Theory | [83] |
| TAM |  |  |
| GDT |  |  |
| PMT |  |  |
| Attitude | Construct/IndependentVariable, PsychoSocialCulturalDemographicVariable |  |
| Sanctions |  |  |
| CopingAppraisal |  |  |
| ThreatAppraisal |  |  |
| AttitudeTowardBehavior |  |  |
| SubjectiveNorm |  |  |
| PerceivedBehavioralControl |  |  |
| PerceivedUsefulness |  |  |
| PerceivedEaseOfUse |  |  |
| PerceivedSeverity |  |  |
| PerceivedCertainty |  |  |
| ResponseEfficacy |  |  |
| SelfEfficacy |  |  |
| ResponseCost |  |  |
| PerceivedSeverityOfThreat |  |  |
| PerceivedVulnerability |  |  |
| BehavioralIntention |  |  |
| ActualBehavior | DependentVariable, SecurityPractice |  |
| Demographic | Construct/IndependentVariable, PsychoSocialCulturalDemographicVariable | [35] |
| Gender |  |  |
| PriorExperience |  |  |
| ComputerSkills |  |  |
| CuesToAction |  |  |
| SecuritySelfEfficacy |  |  |
| SelfReportedCybersecurityBehavior | DependentVariable, SecurityPractice |  |
| HBM | Theory | [49] |
| PerceivedSuspectibility | Construct/IndependentVariable, PsychoSocialCulturalDemographicVariable |  |
| PerceivedBenefits |  |  |
| SelfEfficacy |  |  |
| EmailSecurity | DependentVariable, SecurityPractice |  |
| Unattended asset goes missing | DependentVariable, SecurityPractice | [46] |
| Password or access token sharing |  |  |
| Email to wrong recipient |  |  |
| Theft on premises |  |  |
| Procedure not followed |  |  |
| Wrong privileges set |  |  |
| High-impact mistakes |  |  |
| Working in public place |  |  |
| Unsecure remote third party |  |  |
| Transportation |  |  |
| Family breach |  |  |
| Backup medium goes missing |  |  |
| Improper disposal |  |  |
| Third-party discloses data |  |  |
| Unsecure remote working |  |  |
| Trainee breach |  |  |
| Patient breach |  |  |
| Covering up errors |  |  |
| AttachmentToJob | Construct/IndependentVariable, PsychoSocialCulturalDemographicVariable | [34] |
| AttachmentToOrganization |  |  |
| CoworkerBehavior |  |  |
| SubjectiveNorms |  |  |
| Commitment |  |  |
| PerceivedSeverity |  |  |
| BehavioralIntention | DependentVariable, SecurityPractice |  |
| PerceivedCertaintyOfSanction | Construct/IndependentVariable, PsychoSocialCulturalDemographicVariable | [84] |
| PerceivedSeverityOfSanction |  |  |
| Attitude |  |  |
| BehavioralIntention | DependentVariable, SecurityPractice |  |
| PasswordSharing | DependentVariable, SecurityPractice | [9] |
| AutheticationCircumvention |  |  |
| DeAutheticationCircumvention |  |  |
| PermissionManagement |  |  |
| RepresentationBreaking |  |  |
| Conscientiousness | Construct/IndependentVariable, PsychoSocialCulturalDemographicVariable | [85] |
| Agreeableness |  |  |
| EmotionalStability |  |  |
| RiskTakingPropensity |  |  |
| InformationSecurityAwareness | DependentVariable, SecurityPractice |  |
| PasswordManagement | DependentVariable, SecurityPractice | [37] |
| EmailUse |  |  |
| InternetUse |  |  |
| SocialMediaUse |  |  |
| MobileDeviceUse |  |  |
| InformationHandling |  |  |
| IncidentReporting |  |  |
